# Association between blood culture positivity and in-hospital mortality in pediatric oncology patients with sepsis in the PICU: a retrospective cohort analysis

**DOI:** 10.3389/fcimb.2026.1888168

**Published:** 2026-09-30

**Authors:** Dianwei Xing, Zijing Xiao, Baiming Liu, Jingjing Xu, Yufeng Liang, Xiaoxing Wei

**Affiliations:** Department of Pediatric Critical Care Medicine, Guangzhou Women and Children’s Medical Center, Guangzhou Medical University, Guangzhou, Guangdong, China

**Keywords:** blood culture positivity, mortality, organ dysfunction, pediatric oncology, PICU (pediatric intensive care unit), sepsis

## Abstract

**Background:**

Sepsis is one of the major causes of death in pediatric oncology patients. However, whether blood culture positivity is independently associated with mortality in this high-risk, immunocompromised population remains unclear. We evaluated whether blood culture results are associated with survival outcomes in pediatric oncology patients with sepsis treated in the pediatric intensive care unit (PICU).

**Methods:**

We retrospectively identified sepsis episodes among pediatric oncology patients admitted to the PICU between 2016 and 2024 according to the Phoenix Sepsis Criteria. Clinical variables were compared by blood culture status and in-hospital survival. We used generalized estimating equation (GEE) logistic regression models that accounted for clustering of sepsis episodes within patients to assess the association between blood culture positivity and in-hospital mortality.

**Results:**

The primary analysis included 234 sepsis episodes contributed by 209 patients; all episodes met the Phoenix pediatric sepsis criteria. Blood cultures were positive in 48 episodes (20.5%), and in-hospital death occurred in 45 episodes (19.2%). The blood culture–positive group had higher procalcitonin (PCT) levels (p = 0.015), whereas no statistically significant differences were observed in Phoenix Sepsis Score, organ function indicators, invasive mechanical ventilation (IMV) or continuous renal replacement therapy (CRRT) requirements, PICU length of stay, or in-hospital mortality. In GEE models accounting for within-patient clustering, blood culture positivity was not independently associated with in-hospital mortality (OR = 1.412, 95% CI 0.536–3.719, p = 0.485). In contrast, higher lactate and vasoactive-inotropic score (VIS) were independently associated with increased mortality, whereas higher PaO_2_/FiO_2_ and platelet counts were associated with lower mortality.

**Conclusion:**

In this single-center retrospective cohort of sepsis episodes among pediatric oncology patients, blood culture positivity was not independently associated with in-hospital mortality after adjustment for age, organ dysfunction markers, and prior antimicrobial exposure. In contrast, several markers of organ dysfunction remained independently associated with mortality. However, given the limited number of deaths and the potential for culture-status misclassification related to prior antimicrobial exposure, a clinically meaningful association between blood culture positivity and mortality cannot be excluded.

## 1 Introduction

Sepsis is a major global health challenge and a leading cause of pediatric mortality. In 2017, it accounted for an estimated 49 million cases and roughly 11 million deaths worldwide. Children represented nearly half of these cases, with over 3 million annual deaths, disproportionately affecting resource-limited regions (Rudd et al., 2020; Bassat et al., 2023; Watson et al., 2024). This highly heterogeneous syndrome can complicate a range of underlying conditions. Pediatric oncology patients are particularly vulnerable due to cancer-induced organ infiltration, immune dysregulation, and chemotherapy-associated immunosuppression, neutropenia, and mucosal barrier compromise (Wolf et al., 2013; Soeteman et al., 2023). As a result, children receiving anticancer therapy face a higher risk of sepsis-related death (Aljabari et al., 2019; Rubnitz et al., 2023). Previous studies have reported that mortality among oncology patients hospitalized in the PICU with sepsis approaches 30% (Wösten-van Asperen et al., 2023). Moreover, immune impairment in this population not only increases susceptibility to infection but also delays early recognition, facilitating rapid and often clinically subtle sepsis progression.

Despite advances in antimicrobial therapy, organ support, and critical care management, mortality from sepsis and septic shock remains substantial, ranging from 20% to 40% depending on disease severity (Vincent et al., 2019; Liu et al., 2022). Microbiological assessment plays a pivotal role in the early evaluation and management of sepsis. Blood culture remains the standard method for identifying bloodstream pathogens and guiding antibiotic therapy, with current sepsis survival guidelines recommending culture collection prior to initiating antimicrobial treatment (Prescott et al., 2026). Although microbiological culture is an essential component of early sepsis management, the association between blood culture positivity and mortality remains uncertain. In adult sepsis populations, blood culture–positive patients have been reported to differ in clinical outcomes compared with culture-negative patients (Brooks et al., 2016; Nannan Panday et al., 2019). Recent studies, however, indicate that mortality and ICU length of stay do not differ significantly between blood culture–positive and –negative patients (Paquette et al., 2021; Nejtek et al., 2023). Evidence in pediatric sepsis, particularly among children with malignancies and compromised immunity, is limited (Hazwani et al., 2020). It remains unclear whether blood culture positivity is associated with infection characteristics, organ dysfunction, or clinical outcomes in PICU-admitted pediatric oncology patients. Clarifying this relationship could improve pathogen assessment, risk stratification, and early clinical management in this high-risk population.

Blood cultures are essential for identifying pathogens and guiding antimicrobial therapy. However, whether blood culture positivity remains associated with in-hospital mortality after accounting for established markers of illness severity remains uncertain, especially in immunocompromised pediatric oncology patients. Therefore, the present study evaluated the association between blood culture positivity and in-hospital mortality among pediatric oncology patients requiring PICU admission for sepsis, with particular attention to whether this association persisted after accounting for clinical characteristics and markers of illness severity.

## 2 Methods

### 2.1 Study design

We included pediatric oncology patients admitted to the PICU of Guangzhou Women and Children’s Medical Center for sepsis between January 2016 and December 2024. The PICU is a regional referral center that provides intensive care for children with severe medical and surgical conditions. Sepsis episodes were excluded from the primary analysis if any of the following applied: (1) patient age ≤28 days or ≥18 years; (2) documented death following withdrawal of life-sustaining treatment; (3) discharge against medical advice at the request of guardians; or (4) missing essential data. One episode was excluded because more than 50% of the clinical and laboratory variables required for the prespecified analyses were unavailable. No imputation was performed, and the remaining 234 episodes had complete data for the variables included in the primary analyses. Episodes with documented death following withdrawal of life-sustaining treatment were excluded from the primary analysis because death occurred after a decision to limit life-sustaining treatment. Episodes involving discharge against medical advice were excluded because subsequent vital status after discharge could not be reliably determined. Both withdrawal of life-sustaining treatment and discharge against medical advice were undertaken at the request of the patients’ guardians. Because exclusion of these episodes could nevertheless introduce selection bias related to illness severity or prognosis, their influence was evaluated in sensitivity analyses. These criteria were applied to improve the completeness and reliability of in-hospital outcome data. For children with multiple PICU admissions during the study period, readmission within 48 hours after transfer to a general ward was considered part of the same episode. Readmission after more than 48 hours was considered a new episode. A total of 258 potentially eligible sepsis episodes were identified. Of these, 24 episodes were excluded, including one with missing essential data, 10 involving guardian-directed withdrawal of treatment, and 13 involving discharge against medical advice. The final cohort therefore comprised 234 sepsis episodes contributed by 209 patients. Among these patients, 21 contributed more than one eligible sepsis episode, with a maximum of four episodes contributed by a single patient. The study selection process is shown in **Figure 1**. We retrospectively reviewed patient data retrieved from the institutional electronic medical records platform. All data were anonymized, and repeated episodes from the same patient were linked using a unique anonymized patient identifier. This study received approval from the Ethics Committee of Guangzhou Women and Children’s Medical Center (Approval No. 323A01), with informed consent waived. The research was carried out in accordance with the Declaration of Helsinki (1964) and subsequent amendments or comparable ethical standards. This study was reported in accordance with the Strengthening the Reporting of Observational Studies in Epidemiology (STROBE) guidelines.

**Figure 1:**
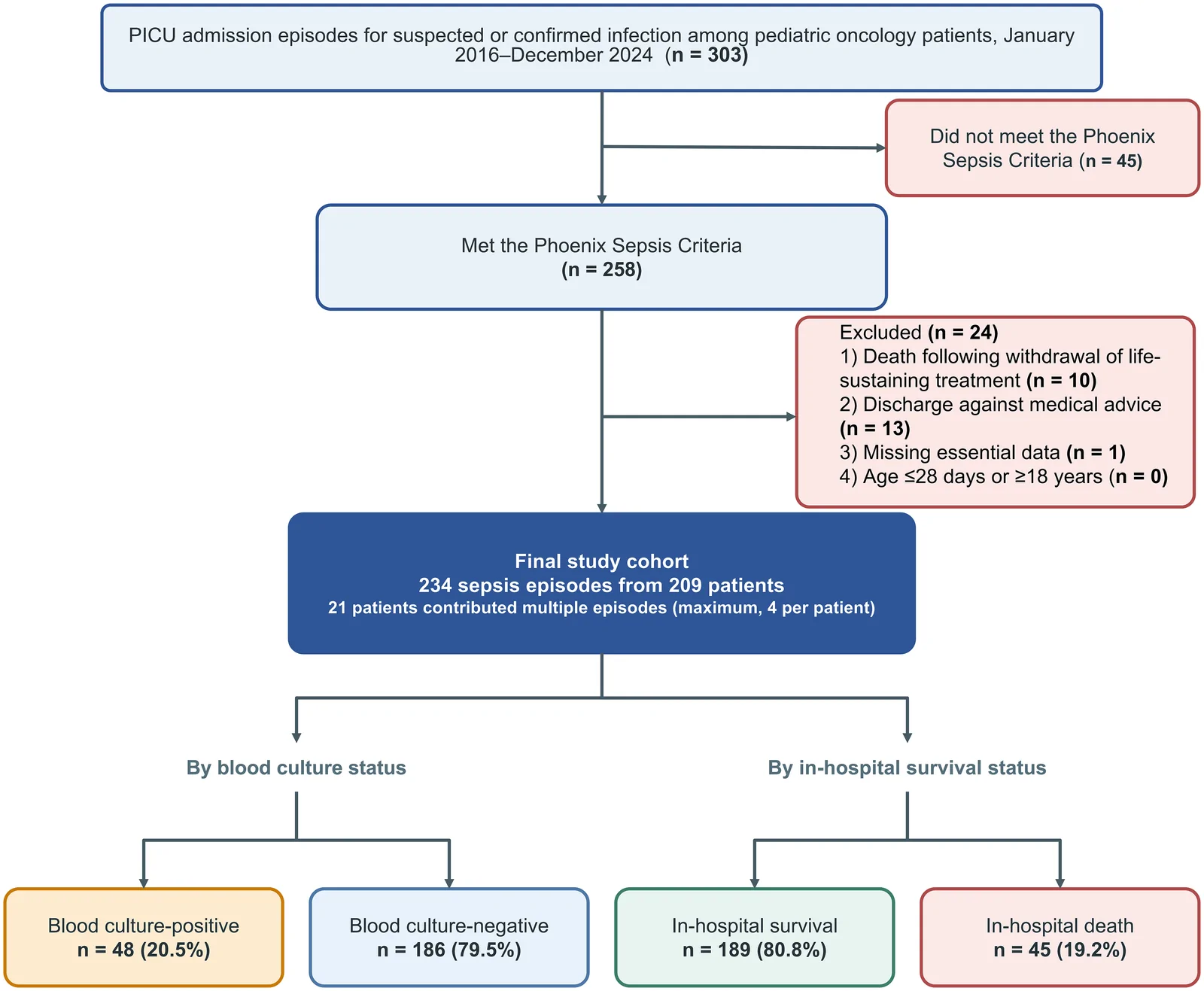
Flowchart of the study.

### 2.2 Outcomes and definitions

Hospital death served as the primary clinical endpoint, while secondary measures included duration of PICU admission as well as utilization of IMV and CRRT. The diagnosis of sepsis was established according to the 2024 Phoenix diagnostic criteria for pediatric sepsis. Under these criteria, a Phoenix Sepsis Score of ≥2 was used to define sepsis in children with either suspected or confirmed infection. Suspected infection was operationally defined as the administration of systemic antimicrobial agents together with laboratory cultures obtained within 24 h of PICU admission, as outlined in the Phoenix Sepsis Score development guidelines (Sanchez-Pinto et al., 2024). Blood culture positivity was defined as the isolation of a pathogenic bacterium or fungus from cultures collected during the predefined index window. This window extended from 72 h before to 48 h after PICU admission. IMV use was defined as invasive mechanical ventilation initiated within the first 24 hours after PICU admission, whereas CRRT use was defined as continuous renal replacement therapy administered at any time during the PICU stay. For common skin commensals, blood culture positivity was considered clinically significant only when at least two independent blood cultures were positive and compatible clinical signs of infection were present (FAQs: Bloodstream Infection (BSI) Events | NHSN | CDC, 2026).

### 2.3 Blood culture collection and microbiological procedures

Blood cultures were obtained as part of routine clinical care when bloodstream infection or sepsis was suspected. According to institutional practice, cultures were collected as early as possible, preferably before the initiation of empirical antimicrobial therapy when clinically feasible. In some patients, particularly those transferred to the PICU after initial antimicrobial treatment, cultures may have been obtained after antibiotic exposure. Samples were obtained from peripheral venipuncture and/or indwelling central venous catheters according to clinical judgment. All cultures were processed by the institutional microbiology laboratory using standard procedures. Due to the retrospective design, detailed information on exact sampling time, sampling site, blood volume, and number of culture sets was not uniformly available for all patients and, therefore, was not included in the primary analysis.

### 2.4 Data collection

Patient information was obtained from the hospital electronic health record database, comprising demographic characteristics, clinical features, and laboratory parameters. The collected data included sex, age, oncological diagnosis, and clinical outcomes (survival or death). PICU-related data included the length of PICU stay, the cause and site of infection, microbiological test results, mean arterial pressure (MAP), as well as respiratory support, vasoactive drug support, and CRRT, among other treatments. In addition, antibiotic exposure data prior to PICU admission were collected for all patients, including whether antibiotics were used before PICU admission, duration of antibiotic exposure prior to PICU admission (categorized as ≤48 h or >48 h), whether combination antibiotic therapy (≥2 antibiotics) was administered, and whether carbapenem antibiotics were used. Laboratory data measured within 24 h after PICU admission included absolute neutrophil count (ANC), creatinine (Cr), aspartate aminotransferase (AST), alanine aminotransferase (ALT), bilirubin, albumin, lactate, platelet, international normalized ratio (INR), fibrinogen (FIB), prothrombin time (PT), PCT, and C-reactive protein (CRP). The VIS was calculated using measurements taken within the initial 24 hours of PICU admission, considering the number and dose of vasoactive agents used. Physiological and laboratory parameters necessary for calculating the Phoenix Sepsis Score were also collected. Phoenix Sepsis Score data were based on information from the initial 24 hours of PICU admission. If a variable had multiple records on the same day, the most extreme value was selected for scoring according to standard operating procedures. To ensure data accuracy and consistency, data entry was performed by designated PICU staff and independently supervised by another staff member. All collected data were verified by senior clinicians.

### 2.5 Statistical analysis

Statistical analyses were performed in R version 4.4.1. Continuous variables are reported as mean (standard deviation) or median (interquartile range), as appropriate, and categorical variables as n (%). Groups were compared using Student’s t-test or the Mann–Whitney U test for continuous variables and the χ² test or Fisher’s exact test for categorical variables. All tests were two-sided, and p < 0.05 was considered statistically significant. For continuous variables in **Table 1**, 95% CIs for median differences were estimated using 5, 000 patient-level cluster bootstrap resamples with the percentile method, retaining all eligible episodes contributed by each sampled patient within the same bootstrap sample. Unadjusted group comparisons were considered descriptive and did not account for within-patient clustering. Blood culture positivity was the primary exposure and in-hospital mortality the primary outcome. GEE logistic regression with a logit link, an exchangeable working correlation structure, and robust sandwich standard errors was used to account for correlation among repeated sepsis episodes within patients, with the anonymized patient identifier specified as the clustering variable. Blood culture positivity was retained in all models. Covariates were prespecified according to clinical relevance and prior evidence rather than selected based on univariable p-values. Given the limited number of deaths, four parsimonious prespecified alternative model specifications, rather than sequentially nested models, were evaluated. Model 1 was unadjusted; Model 2 included age, hematologic malignancy, and neutropenia; Model 3 included lactate, PaO_2_/FiO_2_, platelet count, PCT, and VIS; and Model 4 included age, the Model 3 severity markers, and pre-PICU antimicrobial exposure >48 h. Models 3 and 4 were interpreted as estimating the incremental association of blood culture positivity beyond measured organ dysfunction rather than a direct causal effect. Sensitivity analyses evaluated alternative definitions of antecedent antimicrobial exposure (any pre-PICU use, carbapenem exposure, and combination therapy), restriction to each patient’s first eligible episode, and use of the Phoenix Sepsis Score as an alternative severity measure. To assess the potential influence of excluding episodes involving withdrawal of life-sustaining treatment, an additional analysis retained in-hospital mortality as the outcome and included episodes with documented death following treatment withdrawal; episodes involving discharge against medical advice were excluded from this mortality analysis because subsequent vital status was unavailable. In a separate conservative sensitivity analysis, in-hospital death, documented death following treatment withdrawal, and discharge against medical advice were combined as a composite adverse outcome; discharge against medical advice was treated as an adverse event for this analysis only and was not considered confirmed death. These sensitivity analyses used GEE with patient-level clustering and the corresponding adjusted covariate specification. Variance inflation factors were calculated for Model 4. No formal internal validation procedure was performed because the multivariable models were intended to estimate associations rather than to develop or validate a prediction model.

**Table 1:** Comparison of blood culture-positive and blood culture-negative sepsis episodes.

Variable	All (N = 234)	Blood culture-positive (n = 48)	Blood culture-negative (n = 186)	Effect estimate (95% CI)	p
Phoenix Sepsis Score	4 (3–6)	4 (3–6)	4 (3–6)	0.00 (−1.00 to 1.00)	0.941
PaO_2_/FiO_2_, median (IQR)	195.3 (129.5–276.0)	198 (132.3–252.3)	191.3 (127.9–277.5)	6.75 (−43.50 to 33.50)	0.782
Lactate (mmol/L), median (IQR)	2.0 (1.1–4.3)	2 (1.3–2.6)	2 (1.1–4.4)	0.00 (−0.45 to 0.46)	0.986
MAP (mmHg), median (IQR)	58.0 (51.0–68.0)	58 (51.3–66.0)	58 (50.8–68.0)	0.00 (−4.51 to 5.00)	0.662
VIS, median (IQR)	8.8 (0–30.0)	17.5 (0–36.9)	6.3 (0–30.0)	11.25 (0.00 to 22.50)	0.070
Bilirubin (mg/dL), median (IQR)	0.7 (0.4–1.6)	0.9 (0.4–1.8)	0.7 (0.4–1.4)	0.20 (−0.12 to 0.77)	0.255
ALT (U/L), median (IQR)	22.0 (12.0–47.0)	19.5 (12.0–66.0)	22.0 (12.0–44.0)	−2.50 (−8.40 to 20.00)	0.583
AST (U/L), median (IQR)	44.0 (24.0–97.8)	61.5 (25.8–132.5)	41.5 (24.0–87.8)	20.00 (−10.50 to 55.50)	0.186
Albumin (g/L), median (IQR)	34.0 (29.9–38.5)	31.6 (29.4–38.8)	34.3 (30.3–38.6)	−2.70 (−4.60 to 0.20)	0.141
Creatinine (µmol/L), median (IQR)	28.0 (20.0–43.0)	30.0 (19.5–43.8)	27.0 (20.0–43.0)	3.00 (−3.00 to 11.00)	0.535
FIB (g/L), median (IQR)	2.6 (1.7–3.9)	3.1 (2.0–4.0)	2.5 (1.7–3.9)	0.62 (−0.03 to 1.16)	0.169
INR, median (IQR)	1.3 (1.1–1.6)	1.3 (1.1–1.7)	1.3 (1.1–1.6)	−0.02 (−0.17 to 0.13)	0.790
PT (s), median (IQR)	16.3 (14.5–19.3)	16.2 (14.3–19.8)	16.3 (14.5–19.2)	−0.10 (−1.60 to 1.50)	0.897
CRP (mg/L), median (IQR)	61.3 (20.0–140.3)	98.9 (25.7–140.9)	57.1 (16.9–140.3)	41.80 (−21.08 to 70.13)	0.181
PCT (ng/mL), median (IQR)	1.1 (0.2–10.7)	6.1 (0.4–18.5)	1.0 (0.2–7.7)	5.13 (0.23 to 10.33)	0.015
Platelet count (×10^9^/L), median (IQR)	39 (19.0–87.3)	34 (18.0–91.0)	42.5 (20.5–87.0)	−8.50 (−24.00 to 14.50)	0.659
IMV within 24 h after PICU admission, n (%)	152 (65.0%)	30 (62.5%)	122 (65.6%)	0.87 (0.45–1.69)	0.689
CRRT during PICU stay, n (%)	24 (10.3%)	3 (6.3%)	21 (11.3%)	0.52 (0.15–1.82)	0.426
In-hospital mortality, n (%)	45 (19.2%)	10 (20.8%)	35 (18.8%)	1.14 (0.52–2.50)	0.752
PICU stay (days), median (IQR)	7 (3–13)	9 (3–13)	7 (3–14)	2.00 (−3.00 to 5.00)	0.638

For continuous variables, effect estimates are median differences (blood culture-positive minus blood culture-negative), with 95% CIs derived from 5, 000 patient-level cluster bootstrap resamples using the percentile method. For categorical variables, unadjusted ORs with 95% CIs are reported. Group-comparison p values are descriptive and do not account for within-patient clustering. ALT, alanine aminotransferase; AST, aspartate aminotransferase; CI, confidence interval; CRP, C-reactive protein; CRRT, continuous renal replacement therapy; FIB, fibrinogen; IMV, invasive mechanical ventilation; INR, international normalized ratio; IQR, interquartile range; MAP, mean arterial pressure; OR, odds ratio; PaO_2_/FiO_2_, ratio of arterial oxygen partial pressure to fraction of inspired oxygen; PCT, procalcitonin; PICU, pediatric intensive care unit; PT, prothrombin time; VIS, vasoactive-inotropic score.

## 3 Results

### 3.1 Study population and baseline features

The final analytical cohort comprised 234 sepsis episodes from 209 patients. **Table 2** summarizes the baseline characteristics of the cohort. The median age was 56.5 months (IQR: 28.0–110.8), including 127 males (54.3%) and 107 females (45.7%). Acute lymphoblastic leukemia represented the predominant malignancy (41.9%), with myeloid leukemia (26.5%), lymphoma (17.9%), and solid tumors (13.7%) accounting for the remaining cases. Neutropenia (ANC <500/µL) was observed in 116 episodes (49.6%). The median Phoenix Sepsis Score was 4 (IQR: 3–6). Blood cultures were positive in 48 episodes (20.5%). Invasive mechanical ventilation was required within 24 h of PICU admission in 152 episodes (65.0%). Continuous renal replacement therapy was used during the PICU stay in 24 episodes (10.3%). The median PICU stay duration was 7 days (IQR: 3–13). In-hospital death occurred in 45 sepsis episodes (19.2%). Overall, 220 episodes (94.0%) received antibiotics prior to PICU admission. Prolonged antibiotic exposure (>48 hours before PICU admission) was observed in 149 episodes (63.7%). Combination antibiotic therapy (defined as the use of ≥2 antibiotics) was administered in 168 episodes (71.8%), and carbapenem use was recorded in 183 episodes (78.2%).

**Table 2:** Baseline characteristics of sepsis episodes in pediatric oncology patients.

Variable	Total (N = 234 episodes)
Demographics and malignancy characteristics	
Age, months, median (IQR)	56.5 (28.0–110.8)
Male sex, n (%)	127 (54.3%)
Diagnostic group	
Hematologic, n (%)	202 (86.3%)
Acute lymphoblastic leukemia	98 (41.9%)
Myeloid leukemia	62 (26.5%)
Lymphoma	42 (17.9%)
Solid tumor, n (%)	32 (13.7%)
Neutropenia (ANC <500/µL), n (%)	116 (49.6%)
Sepsis severity and organ dysfunction	
Phoenix Sepsis Score, median (IQR)	4 (3–6)
PaO_2_/FiO_2_, median (IQR)	195.3 (129.5–276.0)
Lactate (mmol/L), median (IQR)	2.0 (1.1–4.3)
MAP (mmHg), median (IQR)	58.0 (51.0–68.0)
VIS, median (IQR)	8.8 (0.0–30.0)
Bilirubin (mg/dL), median (IQR)	0.7 (0.4–1.6)
ALT (U/L), median (IQR)	22.0 (12.0–47.0)
AST (U/L), median (IQR)	44.0 (24.0–97.8)
Albumin (g/L), median (IQR)	34.0 (29.9–38.5)
Creatinine (µmol/L), median (IQR)	28.0 (20.0–43.0)
FIB (g/L), median (IQR)	2.6 (1.7–3.9)
INR, median (IQR)	1.3 (1.1–1.6)
PT (s), median (IQR)	16.3 (14.5–19.3)
CRP (mg/L), median (IQR)	61.3 (20.0–140.3)
PCT (ng/mL), median (IQR)	1.1 (0.2–10.7)
Microbiological characteristics	
Blood culture-positive episodes, n (%)	48 (20.5%)
Antibiotic exposure	
Pre-PICU antibiotic use, n (%)	220 (94.0%)
Pre-PICU antibiotic exposure >48 h, n (%)	149 (63.7%)
Carbapenem use, n (%)	183 (78.2%)
Combination therapy (≥2 antibiotics), n (%)	168 (71.8%)
Organ support and outcomes	
IMV within 24 h after PICU admission, n (%)	152 (65.0%)
CRRT during PICU stay, n (%)	24 (10.3%)
PICU stay (days), median (IQR)	7 (3–13)
In-hospital mortality, n (%)	45 (19.2%)

ALT, alanine aminotransferase; ANC, absolute neutrophil count; AST, aspartate aminotransferase; CRP, C-reactive protein; CRRT, continuous renal replacement therapy; FIB, fibrinogen; IMV, invasive mechanical ventilation; INR, international normalized ratio; IQR, interquartile range; MAP, mean arterial pressure; PCT, procalcitonin; PICU, pediatric intensive care unit; PT, prothrombin time; VIS, vasoactive-inotropic score; PaO_2_/FiO_2_, ratio of arterial oxygen partial pressure to fraction of inspired oxygen.

### 3.2 Microbiological characteristics of blood culture-positive sepsis episodes

Pathogen distribution in episodes with positive blood cultures is depicted in **Figure 2**. Gram-negative bacteria were the predominant isolates, accounting for 69.1%, followed by Gram-positive bacteria (16.4%) and fungi (14.5%). Detailed pathogen distribution is presented in **Figure 3**. The most frequently isolated pathogens were *Klebsiella pneumoniae* (21.8%), *Pseudomonas aeruginosa* (12.7%), *Escherichia coli* (10.9%), and *Candida tropicalis* (7.3%). Among the 48 blood culture-positive sepsis episodes, 6 (12.5%) involved polymicrobial bloodstream infections, whereas the remaining 42 (87.5%) were monomicrobial. In exploratory univariable analysis, in-hospital mortality differed across pathogen subgroups (p = 0.049) (**Table 3**).

**Figure 2:**
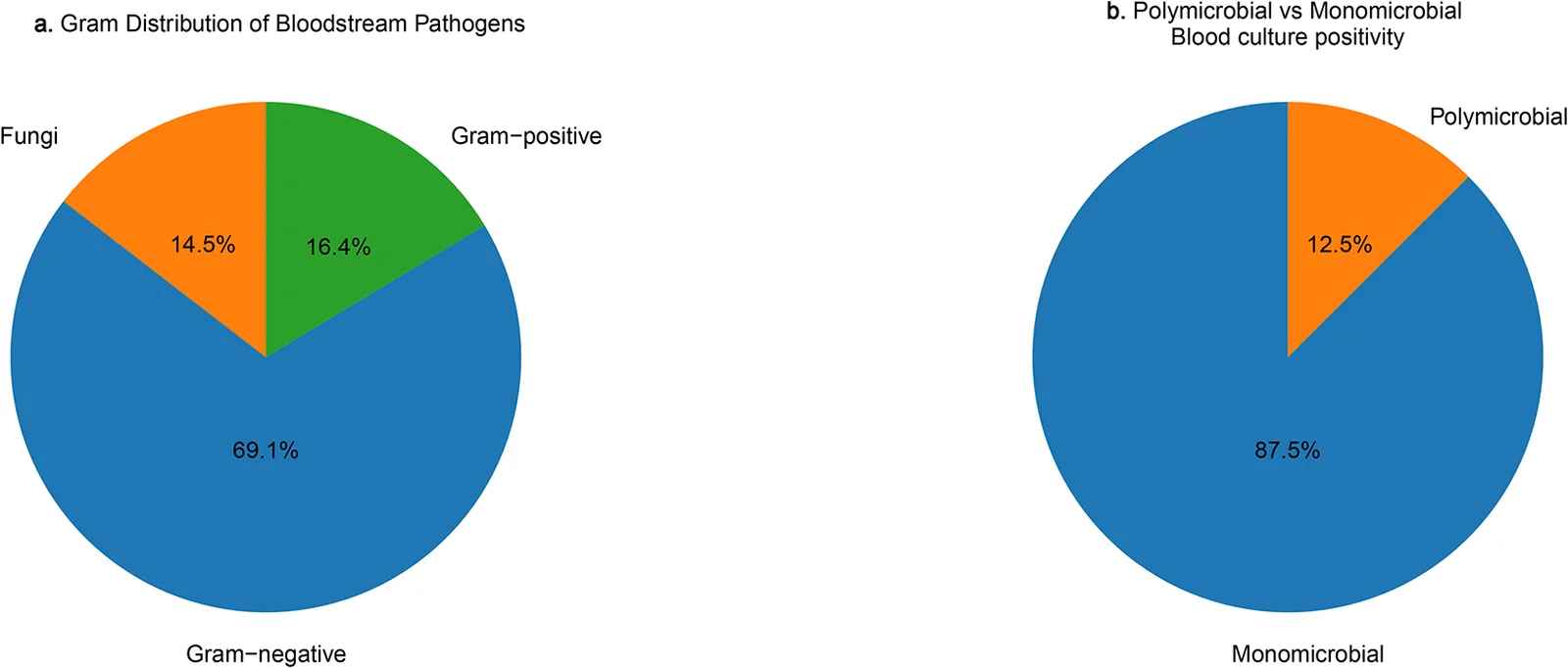
**(a)** Distribution of 55 bloodstream pathogen isolates recovered from 48 blood culture-positive sepsis episodes; **(b)** distribution of monomicrobial and polymicrobial bloodstream infections among the 48 blood culture-positive sepsis episodes.

**Figure 3:**
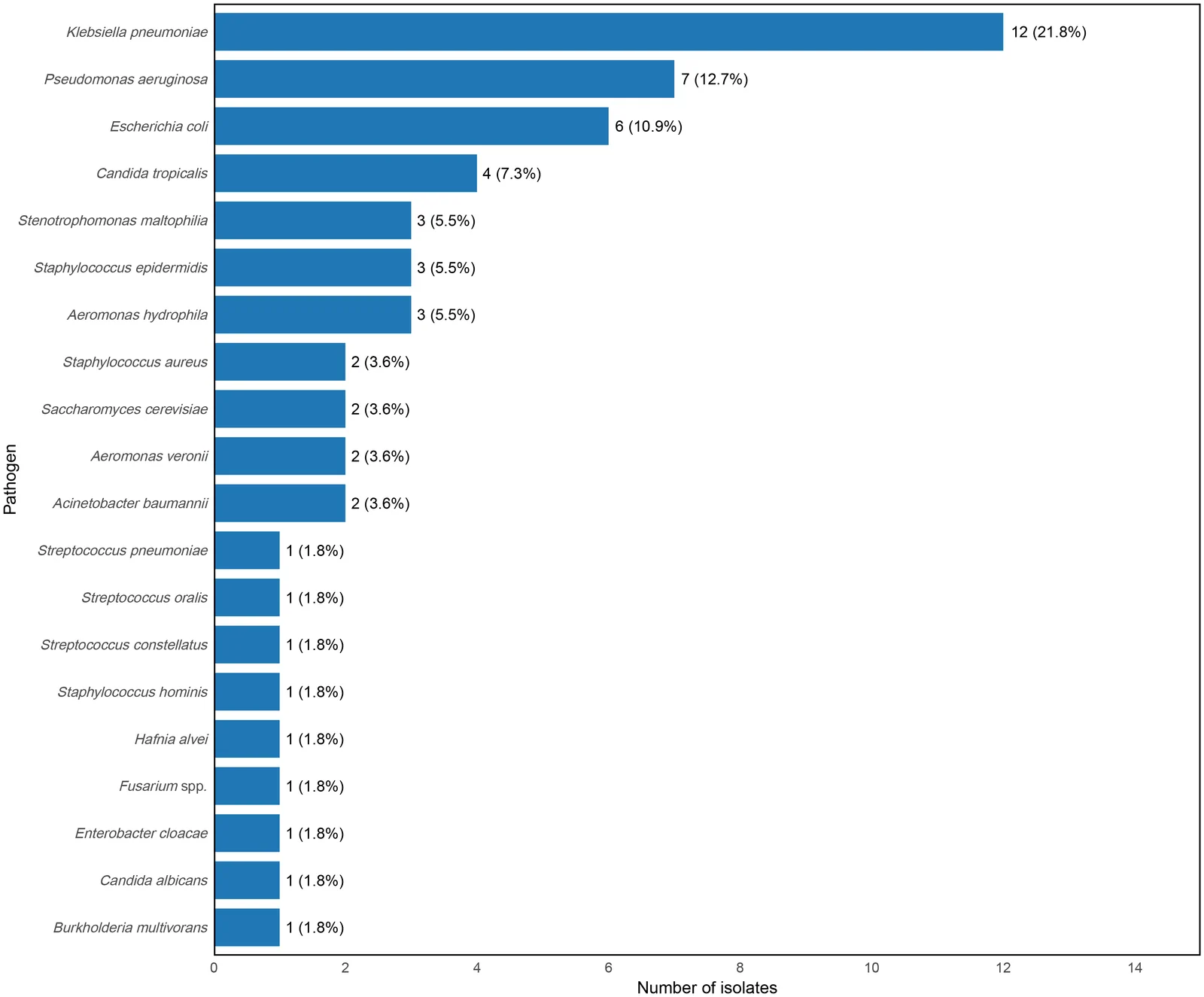
Distribution of 55 pathogen isolates recovered from 48 blood culture-positive sepsis episodes. Percentages were calculated using the total number of isolates (n = 55) as the denominator.

**Table 3:** Univariate analysis of in-hospital mortality by pathogen subgroups.

Pathogen subgroup	n	In-hospital survival, n (%)	In-hospital death, n (%)	p
Overall comparison				0.049
Gram-negative	28	24 (85.7%)	4 (14.3%)	
Gram-positive	9	6 (66.7%)	3 (33.3%)	
Fungi	5	2 (40.0%)	3 (60.0%)	
Polymicrobial	6	6 (100%)	0 (0%)	

Percentages are calculated within each pathogen subgroup. The overall p-value compares the four pathogen categories and should be interpreted as exploratory because of the small subgroup sizes.

### 3.3 Comparison of blood culture-positive and blood culture-negative sepsis episodes

**Table 1** compares clinical and laboratory variables between blood culture-positive and blood culture-negative sepsis episodes. Of the 234 sepsis episodes in the primary analysis, 48 (20.5%) were blood culture-positive and 186 (79.5%) were blood culture-negative. The blood culture–positive group had significantly higher PCT levels than the blood culture–negative group (p = 0.015). No statistically significant differences were observed in Phoenix Sepsis Score, PaO_2_/FiO_2_, lactate, MAP, VIS, platelet count, coagulation parameters, or CRP levels. Regarding organ support, IMV within the first 24 h of PICU admission was used in 30 blood culture-positive episodes (62.5%) and 122 blood culture-negative episodes (65.6%; p = 0.689). Three episodes (6.3%) in the blood culture–positive group received CRRT during PICU stay, versus 21 episodes (11.3%) in the blood culture–negative group (p = 0.426). In addition, no statistically significant differences were observed in in-hospital mortality or PICU length of stay.

### 3.4 Comparison of sepsis episodes by in-hospital survival status

In-hospital death occurred in 45 of 234 sepsis episodes (19.2%). **Table 4** compares the clinical characteristics of episodes with in-hospital survival and those ending in in-hospital death. Compared with episodes with in-hospital survival, episodes ending in in-hospital death occurred at a younger age and had higher PCT levels (p = 0.032) and Phoenix Sepsis Scores (p < 0.001). They also showed more severe organ dysfunction, reflected by lower PaO_2_/FiO_2_ and MAP, higher lactate, VIS, and INR, and lower platelet counts, with all differences reaching statistical significance. In contrast, no significant intergroup difference was observed in blood culture positivity (22.2% vs. 20.1%, p = 0.752). No significant intergroup differences were observed in the prevalence of hematologic malignancies, neutropenic status, or CRRT utilization across episodes with in-hospital survival and episodes ending in in-hospital death groups. Regarding antibiotic exposure, no significant differences were observed between episodes with in-hospital survival and episodes ending in in-hospital death in pre-PICU antibiotic use (p = 0.153), pre-PICU antibiotic exposure >48 h (p = 0.248), carbapenem use (p = 0.746), or combination therapy (≥2 antibiotics) (p = 0.174).

**Table 4:** Comparison of sepsis episodes by in-hospital survival status.

Variable	In-hospital survival (n = 189)	In-hospital death (n = 45)	p
Baseline characteristics			
Age (months), median (IQR)	63 (32–113)	40 (25.5–87.5)	0.040
Hematologic malignancy, n (%)	161 (85.2%)	41 (91.1%)	0.298
Neutropenia (ANC <500/µL), n (%)	91 (48.1%)	25 (55.6%)	0.372
Blood culture-positive, n (%)	38 (20.1%)	10 (22.2%)	0.752
Sepsis severity and organ dysfunction			
Phoenix Sepsis Score	4 (3–5)	7 (5–9)	<0.001
PaO_2_/FiO_2_, median (IQR)	210.0 (155.0–279.0)	99.0 (61.8–194.5)	<0.001
Lactate (mmol/L), median (IQR)	1.9 (1.0–3.3)	3.5 (1.6–13.2)	<0.001
MAP (mmHg), median (IQR)	60.0 (52.0–69.0)	53 (43.5–57.5)	<0.001
VIS, median (IQR)	5 (0–28.8)	25 (5.0–41.3)	0.003
Bilirubin (mg/dL), median (IQR)	0.7 (0.4–1.4)	0.7 (0.4–2.4)	0.135
ALT (U/L), median (IQR)	22 (11–46)	25 (14.5–59)	0.566
AST (U/L), median (IQR)	41 (23.5–94.5)	57 (30.5–109.5)	0.151
Albumin (g/L), median (IQR)	34.2 (29.4–39.0)	33.1 (30.5–37.6)	0.856
Creatinine (µmol/L), median (IQR)	28 (20–40.5)	30 (21.0–66.5)	0.178
FIB (g/L), median (IQR)	2.5 (1.8–4.1)	2.8 (1.5–3.9)	0.496
INR, median (IQR)	1.3 (1.1–1.5)	1.5 (1.1–2.0)	0.009
CRP (mg/L), median (IQR)	56.7 (20.0–131.7)	88.0 (17.6–150.9)	0.399
PCT (ng/mL), median (IQR)	1.0 (0.2–9.0)	3.4 (0.4–25.0)	0.032
Platelet count (×10^9^/L), median (IQR)	44 (24–97)	31 (12–61)	0.003
Antibiotic exposure			
Pre-PICU antibiotic use, n/N (%)	180/189 (95.2%)	40/45 (88.9%)	0.153
Pre-PICU antibiotic exposure >48 h, n/N (%)	117/189 (61.9%)	32/45 (71.1%)	0.248
Carbapenem use, n/N (%)	147/189 (77.8%)	36/45 (80.0%)	0.746
Combination therapy (≥2 antibiotics), n/N (%)	132/189 (69.8%)	36/45 (80.0%)	0.174
Organ support			
IMV within 24 h after PICU admission, n (%)	112 (59.3%)	40 (88.9%)	<0.001
CRRT during PICU stay, n (%)	18 (9.5%)	6 (13.3%)	0.423

Continuous variables are presented as median (interquartile range [IQR]) and categorical variables as n/N (%); percentages use the column total as denominator. Continuous variables were compared with the Mann–Whitney U test and categorical variables with the χ² test or Fisher’s exact test, as appropriate. Abbreviations: ALT, alanine aminotransferase; ANC, absolute neutrophil count; AST, aspartate aminotransferase; CRP, C-reactive protein; CRRT, continuous renal replacement therapy; FIB, fibrinogen; IMV, invasive mechanical ventilation; INR, international normalized ratio; MAP, mean arterial pressure; PaO_2_/FiO_2_, ratio of arterial oxygen partial pressure to fraction of inspired oxygen; PCT, procalcitonin; PICU, pediatric intensive care unit; VIS, vasoactive-inotropic score.

### 3.5 GEE logistic regression analysis of in-hospital mortality with adjustment for within-patient clustering

We evaluated four prespecified alternative GEE logistic regression model specifications, all accounting for within-patient clustering (**Table 5**). Blood culture positivity was not statistically significantly associated with in-hospital mortality in the unadjusted model (Model 1: OR = 1.161, 95% CI 0.542–2.489, p = 0.701) or in the baseline-characteristics model (Model 2: OR = 1.319, 95% CI 0.586–2.968, p = 0.503). In the organ-dysfunction model (Model 3), blood culture positivity remained not statistically significantly associated with mortality (OR = 1.316, 95% CI 0.513–3.374, p = 0.568). Similar findings were observed in Model 4, which included age, organ dysfunction markers, and pre-PICU antimicrobial exposure >48 h (OR = 1.412, 95% CI 0.536–3.719, p = 0.485). In Model 4, higher lactate and VIS were independently associated with higher mortality, whereas higher PaO_2_/FiO_2_ and platelet counts were independently associated with lower mortality. No substantial multicollinearity was detected in Model 4 (variance inflation factor range, 1.068–1.138; **Supplementary Table S1**). In an exploratory analysis restricted to blood culture-positive episodes, no statistically significant association with mortality was observed for Gram-negative versus non-Gram-negative infection after adjustment for lactate (**Supplementary Table S2**).

**Table 5:** GEE logistic regression analysis of factors associated with in-hospital mortality, accounting for within-patient clustering.

Variable	OR (95% CI)	p
Model 1. Unadjusted model		
Blood culture positivity	1.161 (0.542–2.489)	0.701
Model 2. Baseline-characteristics model		
Blood culture positivity	1.319 (0.586–2.968)	0.503
Age (months)	0.992 (0.984–1.001)	0.083
Hematologic malignancy	1.999 (0.614–6.513)	0.250
Neutropenia (ANC <500/µL)	1.294 (0.657–2.549)	0.456
Model 3 Illness-severity and organ-dysfunction model		
Blood culture positivity	1.316 (0.513–3.374)	0.568
Lactate	1.207 (1.093–1.334)	<0.001
PaO₂/FiO₂	0.990 (0.985–0.996)	0.001
Platelet count	0.994 (0.989–0.999)	0.014
VIS	1.006 (1.002–1.009)	0.005
PCT	0.995 (0.980–1.010)	0.508
Model 4 Extended organ-dysfunction model		
Blood culture positivity	1.412 (0.536–3.719)	0.485
Age (months)	0.996 (0.986–1.005)	0.362
Lactate	1.226 (1.100–1.366)	<0.001
PaO₂/FiO₂	0.991 (0.985–0.996)	0.001
Platelet count	0.994 (0.990–0.999)	0.024
VIS	1.005 (1.001–1.009)	0.006
PCT	0.995 (0.980–1.011)	0.547
Pre-PICU antibiotic exposure >48 h	1.621 (0.656–4.007)	0.295

GEE models used an exchangeable working correlation structure and robust standard errors, with the anonymized patient identifier as the clustering variable. The four models represent alternative prespecified adjustment specifications and were not sequentially nested. Model 1 included blood culture positivity only. Model 2 included blood culture positivity, age, hematologic malignancy, and neutropenia. Model 3 included blood culture positivity, lactate, PaO₂/FiO₂, platelet count, VIS, and PCT. Model 4 included blood culture positivity, age, the Model 3 severity measures, and pre-PICU antimicrobial exposure >48 h. Values are odds ratios (ORs) with 95% confidence intervals (CIs). Abbreviations: ANC, absolute neutrophil count; CI, confidence interval; GEE, generalized estimating equation; OR, odds ratio; PaO₂/FiO₂, ratio of arterial oxygen partial pressure to fraction of inspired oxygen; PCT, procalcitonin; PICU, pediatric intensive care unit; VIS, vasoactive-inotropic score.

### 3.6 Sensitivity analyses of the association between blood culture positivity and clinical outcomes

Sensitivity analyses using alternative definitions of pre-PICU antimicrobial exposure yielded results similar to those of the primary analysis (**Supplementary Table S3**). The association was also similar when the analysis was restricted to each patient’s first eligible sepsis episode (**Supplementary Table S4**). To assess the influence of excluding treatment-withdrawal-related deaths, the adjusted analysis was repeated after including the 10 episodes with documented in-hospital death following withdrawal of life-sustaining treatment. This analysis included 244 sepsis episodes from 218 patients, with 55 deaths. Blood culture positivity showed no statistically significant association with in-hospital mortality (OR = 1.417, 95% CI 0.582–3.451, p = 0.443). In a separate conservative analysis including all 257 sepsis episodes from 230 patients, 68 composite adverse-outcome events were identified. Blood culture positivity was likewise not statistically significantly associated with the composite outcome (OR = 1.234, 95% CI 0.588–2.590, p = 0.579; **Supplementary Table S5**). Substituting the Phoenix Sepsis Score for the individual organ dysfunction measures produced a similar estimate for blood culture positivity (OR = 1.418, 95% CI 0.494–4.071, p = 0.516). The Phoenix Sepsis Score itself was associated with in-hospital mortality (OR = 2.088, 95% CI 1.664–2.621, p < 0.001; **Supplementary Table S6**).

## 4 Discussion

In this study, we evaluated the association between blood culture positivity and in-hospital mortality. After adjustment for age, markers of organ dysfunction, and antimicrobial exposure before PICU admission, blood culture positivity was not independently associated with in-hospital mortality. The wide confidence interval nevertheless means that a clinically meaningful incremental association cannot be excluded. By contrast, several markers of organ dysfunction remained independently associated with in-hospital mortality.

The blood culture positivity rate in our cohort was lower than the rates of 28%–49% reported in previous studies (Kumar et al., 2009; Rannikko et al., 2017; Rhodes et al., 2017). Several factors may explain this difference. First, empirical broad-spectrum antimicrobial therapy is often initiated promptly when infection is suspected in pediatric oncology patients. When administered before blood sampling, such treatment may reduce blood culture yield (Wang, 2021). Second, blood culture yield is often low in immunocompromised patients, especially those with chemotherapy-induced neutropenia (Rosen and Stohs, 2024). Neutropenia was present in 49.6% of the sepsis episodes in our cohort and may therefore have contributed to the low positivity rate. Blood culture yield also depends on patient characteristics, immune status, the timing and volume of blood collection, the number of culture bottles, and the detection method. Direct comparisons of blood culture positivity rates across studies should therefore be interpreted with caution.

Evidence on the association between blood culture positivity and clinical outcomes in sepsis remains mixed. Several adult studies associated positive blood cultures or bacteremia with higher mortality and longer ICU or hospital stays (Brooks et al., 2016; Nannan Panday et al., 2019). One possible explanation is that culture positivity may reflect a greater pathogen burden or a more invasive infection. More recent studies, however, have reported different findings. Paquette et al. found no significant association between blood culture positivity and mortality (Paquette et al., 2021). Similarly, Nejtek et al. found no statistically significant differences in mortality or ICU length of stay between bacteremic and nonbacteremic patients (Nejtek et al., 2023). These findings are consistent with ours. Evidence in children remains limited. In one PICU cohort of children with severe sepsis or septic shock, mortality was higher in the culture-positive group (Hazwani et al., 2020), in contrast to our findings. The variation across studies may reflect changes in sepsis definitions and diagnostic criteria, differences in patient populations and immune status, variation in microbiological testing protocols, and regional disparities in healthcare resources.

In this cohort, no statistically significant differences were observed between blood culture-positive and blood culture-negative episodes in overall illness severity or the measured indicators of organ dysfunction. Blood culture positivity was not significantly associated with in-hospital mortality, even after adjustment for markers of organ dysfunction. Several factors may explain this finding. Immunosuppression, neutropenia, and antimicrobial exposure before sampling may limit pathogen detection by blood culture (Villeneuve and Aftandilian, 2022; Lehrnbecher et al., 2023; Yang et al., 2023). Some patients with occult bloodstream infection may therefore have been classified as culture-negative. The culture-negative group may also have included patients with severe localized infections. In addition, pediatric oncology patients often receive antimicrobial prophylaxis and prompt empiric treatment for suspected infection (Baden et al., 2024). Early treatment and PICU organ support may have reduced differences in mortality between the two groups. Short-term outcomes in sepsis are primarily associated with dysregulated host responses and consequent organ injury, including circulatory failure, oxygenation deficits, and microcirculatory impairment, rather than the presence of detectable pathogens alone (Singer et al., 2016; Caraballo and Jaimes, 2019; Singer et al., 2026). Blood culture status and organ dysfunction may therefore reflect distinct dimensions of sepsis. Limited culture sensitivity and the resulting misclassification may have biased the association with mortality toward the null.

Markers of host response and organ dysfunction reflect aspects of illness severity that are distinct from pathogen detection by blood culture. In this cohort, PCT levels differed between blood culture-positive and blood culture-negative episodes. PCT levels were also higher in episodes ending in in-hospital death than in episodes with in-hospital survival in univariable analysis. However, PCT was not independently associated with in-hospital mortality after adjustment for clinical severity variables. PCT may therefore reflect infection-related inflammation or the microbiological characteristics of the infection. In contrast, several markers of organ dysfunction remained associated with in-hospital mortality. These findings suggest that short-term outcomes in pediatric oncology patients with sepsis may be more closely related to the extent of organ dysfunction than to whether a bloodstream pathogen is detected.

These findings may inform the clinical assessment and management of pediatric oncology patients with sepsis. Blood cultures remain an essential component of sepsis care. They provide microbiological information for pathogen identification and susceptibility-guided antimicrobial therapy. In immunocompromised patients, however, culture results should be interpreted within the broader clinical context. This is particularly important when clinical evidence of infection persists despite negative cultures. Serial assessment of organ function provides complementary information on the patient’s physiological status and may help identify the need for timely organ support. Clinical management should therefore integrate blood culture results with serial organ function assessment. Together, these approaches provide distinct but complementary microbiological and physiological information to support clinical judgment and treatment decisions.

## 5 Limitations

This study has several limitations. First, the single-center retrospective design may have introduced selection and information bias and limits generalizability. Excluding episodes involving withdrawal of life-sustaining treatment or discharge against medical advice may have introduced additional selection bias related to illness severity or prognosis, although sensitivity analyses yielded similar estimates. Some patients contributed more than one eligible sepsis episode, with the episode used as the unit of analysis. Although GEE were used to account for within-patient clustering, dependence among recurrent episodes may not have been fully captured. Second, only 45 in-hospital deaths occurred, limiting the precision of the multivariable estimates. Candidate variables were prespecified based on clinical relevance, and model complexity was restricted. Nevertheless, overfitting and unstable effect estimates cannot be excluded. The wide confidence intervals for some estimates further indicate uncertainty, and clinically meaningful associations may therefore have been missed. Third, information on the timing of blood culture collection relative to antimicrobial exposure, the number of culture sets, the volume of blood collected, and the sampling site was incomplete. This may have led to misclassification of culture status and reduced the ability to detect associations with clinical outcomes. Fourth, although the analyses included neutropenia and prior antimicrobial exposure, data on central venous catheter status, transplant history, and the severity and duration of neutropenia were incomplete. Residual confounding is therefore possible. Finally, the pathogen-stratified analyses were exploratory. The small number of deaths within each subgroup precluded adequate multivariable adjustment, and these findings should be interpreted cautiously.

## 6 Conclusion

In conclusion, this single-center retrospective cohort study found no independent association between blood culture positivity and in-hospital mortality. However, the limited number of deaths, frequent antimicrobial exposure before blood culture collection, and possible misclassification of culture status mean that a clinically meaningful association cannot be excluded. Blood culture results and organ function assessments capture different dimensions of sepsis and may together provide a more complete assessment of illness severity and clinical risk.

## Data Availability

The raw data supporting the conclusions of this article will be made available by the authors, without undue reservation.
