# Supplementary material for: Association between blood culture positivity and in-hospital mortality in pediatric oncology patients with sepsis in the PICU: a retrospective cohort analysis
Source: Front Cell Infect Microbiol. 2026 Sep 30;16:1888168. doi: 10.3389/fcimb.2026.1888168 (PMC13661487; doi:10.3389/fcimb.2026.1888168)
Supplement: Supplementary file 1 [file Table1.docx]

**Supplementary Material**

**Supplementary Table S1.** Assessment of multicollinearity among variables included in Model 4

| **Variable** | **VIF** |
| --- | --- |
| Blood culture positivity | 1.073 |
| Age (months) | 1.087 |
| Lactate | 1.112 |
| PaO₂/FiO₂ | 1.100 |
| Platelet count | 1.070 |
| VIS | 1.082 |
| PCT | 1.138 |
| Pre-PICU antibiotic exposure> 48 h | 1.068 |

This assessment was based on the primary analytical cohort of 234 sepsis episodes contributed by 209 patients, including 45 in-hospital deaths. Variance inflation factors (VIFs) were calculated for all variables included in Model 4: blood culture positivity, age, lactate, PaO₂/FiO₂, platelet count, VIS, PCT, and pre-PICU antibiotic exposure >48 h. Because VIF is a diagnostic measure of collinearity among covariates rather than an outcome regression analysis, patient-level clustering is not applicable to its calculation. VIF, variance inflation factor; PaO₂/FiO₂, ratio of arterial oxygen partial pressure to fraction of inspired oxygen; PCT, procalcitonin; PICU, pediatric intensive care unit; VIS, vasoactive-inotropic score.

**Supplementary Table S2.** Exploratory analysis of pathogen subgroups among blood culture-positive sepsis episodes

| **Variable** | **OR (95% CI)** | **p** |
| --- | --- | --- |
| Lactate | 1.306 (1.070–1.596) | 0.009 |
| Pathogen group (Gram-negative vs non-Gram-negative) | 0.245 (0.051–1.169) | 0.078 |

This exploratory analysis included 48 blood culture-positive sepsis episodes contributed by 47 patients, with 10 in-hospital deaths; one patient contributed two blood culture-positive episodes. GEE logistic regression was performed using a logit link, an exchangeable working correlation structure, and robust standard errors, with patient identifier specified as the clustering variable to account for within-patient correlation. The model included pathogen group (Gram-negative vs non-Gram-negative) and lactate. The reference group was non-Gram-negative infection. OR, odds ratio; CI, confidence interval; GEE, generalized estimating equatio

**Supplementary Table S3.** Sensitivity analyses using alternative definitions of antecedent antimicrobial exposure and association between blood culture positivity and in-hospital mortality

| **Sensitivity analysis** | **Blood culture positivity OR (95% CI)** | **p** |
| --- | --- | --- |
| Model 1: Any pre-PICU antibiotic use | 1.451 (0.562–3.745) | 0.442 |
| Model 2: Carbapenem exposure | 1.392 (0.525–3.693) | 0.506 |
| Model 3: Combination antibiotic therapy (≥2 antibiotics) | 1.256 (0.448–3.519) | 0.665 |

This analysis included the primary analytical cohort of 234 sepsis episodes contributed by 209 patients, with 45 in-hospital deaths. The sepsis episode was the unit of analysis. GEE logistic regression models used a logit link, an exchangeable working correlation structure, and robust standard errors, with patient identifier specified as the clustering variable. Each model included blood culture positivity, age, PaO₂/FiO₂, lactate, platelet count, VIS, PCT, and the corresponding definition of antecedent antimicrobial exposure (any pre-PICU antibiotic use, carbapenem exposure, or combination antibiotic therapy [≥2 antibiotics]). OR, odds ratio; CI, confidence interval; GEE, generalized estimating equation; PaO₂/FiO₂, ratio of arterial oxygen partial pressure to fraction of inspired oxygen; PCT, procalcitonin; PICU, pediatric intensive care unit; VIS, vasoactive-inotropic score.

**Supplementary Table S4.** Sensitivity analysis restricted to the first eligible sepsis episode per patient

| **Variable** | **OR (95% CI)** | **p** |
| --- | --- | --- |
| Blood culture positivity | 1.303 (0.465–3.646) | 0.614 |
| Age (months) | 0.998 (0.989–1.008) | 0.722 |
| Lactate | 1.228 (1.091–1.383) | <0.001 |
| PaO₂/FiO₂ | 0.991 (0.986–0.996) | 0.001 |
| Platelet count | 0.992 (0.985–0.999) | 0.028 |
| VIS | 1.006 (1.001–1.012) | 0.014 |
| PCT | 0.987 (0.964–1.010) | 0.268 |
| Pre-PICU antibiotic exposure> 48 h | 1.810 (0.693–4.725) | 0.226 |

This analysis was restricted to the first eligible sepsis episode for each of 209 patients and included 41 in-hospital deaths. Because each patient contributed only one episode, observations were independent at the patient level and a conventional multivariable logistic regression model was used. The model included blood culture positivity, age, lactate, PaO₂/FiO₂, platelet count, VIS, PCT, and pre-PICU antibiotic exposure >48 h. OR, odds ratio; CI, confidence interval; PaO₂/FiO₂, ratio of arterial oxygen partial pressure to fraction of inspired oxygen; PCT, procalcitonin; PICU, pediatric intensive care unit; VIS, vasoactive-inotropic score.

**Supplementary Table S5.** Sensitivity analyses addressing episodes excluded because of withdrawal of life-sustaining treatment or discharge against medical advice

| **Variable** | **In-hospital mortality*** |  | **Composite adverse outcome†** |  |
| --- | --- | --- | --- | --- |
|  | OR (95% CI) | p | OR (95% CI) | p |
| Blood culture positivity | 1.417 (0.582–3.451) | 0.443 | 1.234 (0.588–2.590) | 0.579 |
| Age, months | 0.993 (0.984–1.002) | 0.141 | 0.990 (0.982–0.998) | 0.019 |
| Lactate | 1.161 (1.069–1.261) | <0.001 | 1.143 (1.063–1.228) | <0.001 |
| PaO₂/FiO₂ | 0.993 (0.989–0.997) | <0.001 | 0.995 (0.992–0.998) | 0.002 |
| Platelet count | 0.993 (0.989–0.998) | 0.007 | 0.996 (0.992–1.000) | 0.034 |
| VIS | 1.003 (0.999–1.007) | 0.105 | 1.001 (0.997–1.005) | 0.626 |
| PCT | 1.000 (0.984–1.016) | 0.987 | 1.004 (0.992–1.016) | 0.512 |
| Pre-PICU antibiotic exposure> 48 h | 1.264 (0.593–2.694) | 0.544 | 1.354 (0.711–2.579) | 0.356 |

*** In-hospital mortality:** This analysis included 244 sepsis episodes contributed by 218 patients, comprising the 234 episodes in the primary cohort and 10 additional episodes with documented in-hospital death following withdrawal of life-sustaining treatment. A total of 55 in-hospital deaths were included. Episodes involving discharge against medical advice were excluded because subsequent vital status was unavailable.

**†Composite adverse outcome:** This conservative sensitivity analysis included 257 sepsis episodes contributed by 230 patients, with 68 composite adverse-outcome events. The composite outcome was defined as in-hospital death, documented death following withdrawal of life-sustaining treatment, or discharge against medical advice. Discharge against medical advice was classified as an adverse event for this sensitivity analysis only and should not be interpreted as confirmed death.

For both analyses, generalized estimating equation logistic regression was performed using a logit link, an exchangeable working correlation structure, and robust standard errors, with patient identifier as the clustering variable to account for within-patient correlation among repeated sepsis episodes. Models included blood culture positivity, age, lactate, PaO₂/FiO₂, platelet count, VIS, PCT, and pre-PICU antibiotic exposure >48 h.

**Abbreviations:** CI, confidence interval; GEE, generalized estimating equation; OR, odds ratio; PaO₂/FiO₂, ratio of arterial oxygen partial pressure to fraction of inspired oxygen; PCT, procalcitonin; PICU, pediatric intensive care unit; VIS, vasoactive-inotropic score.

**Supplementary Table S6.** Sensitivity analysis incorporating Phoenix Sepsis Score into the multivariable model

| **Variable** | **OR (95% CI)** | **p** |
| --- | --- | --- |
| Blood culture positivity | 1.418 (0.494–4.071) | 0.516 |
| Age (months) | 0.997 (0.987–1.007) | 0.501 |
| Phoenix Sepsis Score | 2.088 (1.664–2.621) | <0.001 |
| PCT | 1.005 (0.990–1.021) | 0.503 |
| Pre-PICU antibiotic exposure> 48 h | 1.848 (0.774–4.409) | 0.167 |

This analysis included the primary analytical cohort of 234 sepsis episodes contributed by 209 patients, with 45 in-hospital deaths. The sepsis episode was the unit of analysis. GEE logistic regression was performed using a logit link, an exchangeable working correlation structure, and robust standard errors, with patient identifier specified as the clustering variable. The model included blood culture positivity, age, Phoenix Sepsis Score, PCT, and pre-PICU antibiotic exposure >48 h; the Phoenix Sepsis Score replaced the individual organ dysfunction measures used in Model 4. OR, odds ratio; CI, confidence interval; GEE, generalized estimating equation; PCT, procalcitonin; PICU, pediatric intensive care unit.
